# Spatial Coupling and Causal Effects between the Recreational Use of Ecological Land and Restoration: A Case Study of the Pearl River Delta Urban Agglomeration

**DOI:** 10.3390/ijerph181910124

**Published:** 2021-09-26

**Authors:** Fuyuan Wang, Rundong Feng

**Affiliations:** 1 Institute of Geographic Sciences and Natural Resources Research, Key Laboratory of Regional Sustainable Development Modeling, Chinese Academy of Sciences, Beijing 100101, China; fengrd.18s@igsnrr.ac.cn; 2 College of Resources and Environment, University of Chinese Academy of Sciences, Beijing 100049, China

**Keywords:** Pearl River Delta urban agglomeration, ecological land restoration, recreational use, ecological recreation spaces, spatial coupling

## Abstract

As the urbanization and industrialization of China’s urban agglomerations reach increasingly high levels, residents are voicing a growing demand for improved green public sport and recreational space. The coordination of ecological land restoration (ELR) and recreational use at the regional level is therefore urgent. This study demonstrates the spatiotemporal evolution of coupled ELR and the recreational use of ecological land (RUoEL) in the Pearl River Delta urban agglomeration based on spatial interpretation, remote sensing mapping, and spatial statistical analysis. A geographical and temporally weighted regression is used to test the spatial effects of the RUoEL on the evolution of the ELR patterns. The results show that the RUoEL (mainly greenways and ecological recreational spaces) and ELR exert a certain degree of coupled spatial characteristics, and that the former significantly impacts the latter. These spatial differences are more notable in areas with high-level ecological recreational spaces, or which are located near densely populated built-up areas. Recreation-oriented ELR is therefore relatively easy to develop in these areas. The results provide important guidelines for the development of ecosystem service patterns in urban agglomerations that include the coexistence of ELR and recreational use, which will strengthen the academic support for regional ELR planning and improve public health.

## 1. Introduction

The recreational use of ecological land (RUoEL) is an approach used to construct green spaces or parks, which generally has a positive effect on public health such as stress relief, fatigue recovery, and fetal and children’s health [1]. Ecological land restoration (ELR) refers to the gradual restoration of a damaged ecosystem or ecosystem development in an environmentally beneficial direction by applying artificial measures based on the ecosystem’s self-recovery ability [2]. Early ELR practices mainly used biological and engineering technology to restore an ecosystem’s basic functions to the original levels or higher [3]. Additionally, conservation biologists often regard recreational activities and tourism as threats to ecological protection [4]. The socioeconomic benefits of restoration have not been emphasized [5], although they may be significant [6]. With the development of urban society, the increasingly popular welfarist view suggests that recreation should be a part of social citizenship [7], and the leisure supply should be guaranteed in the urban public health service system [8]. Especially, the relationship between ecosystems and recreational well-being is increasingly valued. Consequently, ecological restoration and welfarist recreational concepts have spread globally, and tend to merge with each other. In many cities, the potential for human benefit (e.g., aesthetics, recreation) has become one of the driving forces for ELR [9]. In this context, an empirical examination of the spatial interaction between ELR and RUoEL in an urban agglomeration will provide insights into regionally environmental health planning to enhance the recreational well-being of residents [10,11,12,13].

ELR and RUoEL are generally compatible and complementary [14]. Their coordinated advancement in suitable areas is therefore a form of multi-functional land use, and is an important symbol of land use transitions in urbanized areas. ELR practices under the direction of recreation based on landscape design have gradually emerged since the end of the 1990s, especially as an important guide for the ecological restoration of abandoned land. The recreational and touristic use of ecological restoration areas enable people to benefit from cultural services, such as aesthetic appreciation or spiritual enrichment, which offers a good motivation for society to participate in ecological protection and restoration [15]. Urban parks and urban river restoration are cases of ecological improvement strategies usually adopted for social benefit [16]. Multiple cultural service benefits are positively correlated with tourism revenue in the areas with ecological restoration programs [17]. This suggests that the restoration of urban ecosystems should properly take into account social-cultural (e.g., recreation) and economic values [18,19]. A combination of ELR and recreation has thus become an increasingly popular topic in academia.

Specifically, the spatial layout of local- and site-scale ELR and recreational use has gathered extensive research attention. In recent years, the models, specific strategies and concepts of ecological restoration and recreation development based on local-scale ecological protection practices have been discussed widely [20,21,22]. Stanford et al. [9] indicated that the distribution of ELR sites for human benefits is concentrated in areas near perennial streams and population centers, and areas with a high proportion of rich, highly educated, non-Hispanic white people. Site-scale research mainly involves the ecological restoration of abandoned land and the ecological security patterns of restored land under the direction of recreation [23]. For example, Wang et al. [24] proposed to build a coupled economic–environmental tourism system in abandoned mining areas to promote ecological land reconstruction. The second strand of research focused on evaluations of the recreational or cultural service value of a landscape after ELR using evaluation models of cultural ecosystem services [25]. Allan et al. [17] showed that recreational ecosystem services in the ELR area are widely but unevenly distributed, and sites with high cultural service value also have greater environmental pressures. Pueyo-Ros et al. [14] found that ecological restoration had no adverse effect on the recreational value of a wetland. Moreover, the enhancement of the natural value of a tourist destination after ELR will increase the attractiveness of the destination to tourists. Dou et al. [26] reported that the ecological restoration of agricultural landscapes changed the local residents’ perception of the cultural ecosystem services. Westling et al. [27] indicated that the perceived value of local residents before and after ELR is influenced by scenic beauty, the cleanliness of the riverine environment, and the access availability of the river, etc. Loures et al. [28] found that 95% of residents near an industrial wasteland believed that the restored ecological recreational space would improve their quality of life, but different age groups show different preferences for ecological recreation.

Previous studies on ELR and RUoEL have gradually upgraded from the site scale to larger spatial areas to improve the regional ecosystem services. The perspective has shifted from the restoration of the structure and function of an ecosystem to comprehensive restoration through the integration of social culture and economic values. The recreational use patterns and recreational value evaluations of ecological restoration areas have become research hotspots. However, studies have mainly involved the city and site scales. Urban agglomeration areas, characterized by continuous urban expansion and highly integrated ecosystem services, still remain scarcely explored. Especially, there is a lack of empirical evidence on how ELR and ecological recreation spaces achieve spatial evolution and integration on a regional scale.

The Pearl River Delta urban agglomeration (PRDUA) provides an appropriate case study for addressing the above-mentioned issues. First, it is one of the mega-regions with the highest levels of urbanization and regional integration in China. Rapid urbanization and economic development in the PRDUA since the end of the 1980s have resulted in the reduction and fragmentation of urban ecological land, as well as a notable decline of leisure quality. Second, Guangdong Province and its municipalities have implemented land greening activities in the PRDUA since the 1980s, which have promoted an increase of urban forests and urban natural parks, resulting in a noteworthy regional integration of ELR and recreational use. Therefore, our study selected the PRDUA as the case area to investigate the spatial coupling characteristics of ELR and the RUoEL, and to examine the impact of the RUoEL dynamics on ELR. The results provide insights into the coordinated spatial allocation of urban ELR and recreational use to address critical issues related to environmental quality and public health.

## 2. Materials and Methods

### 2.1. Study Area

**Figure 1:**
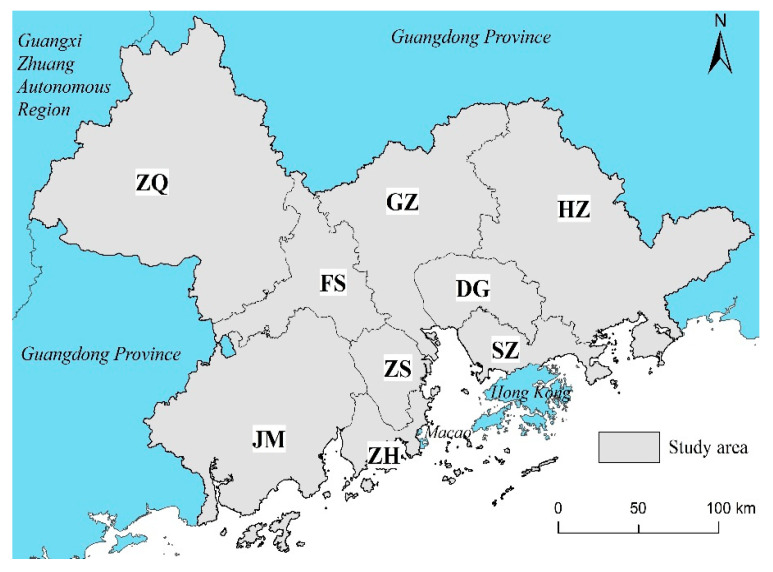
The Pearl River Delta urban agglomeration. ZQ: Zhaoqing; GZ: Guangzhou; FS: Foshan; JM: Jiangmen; ZS: Zhongshan; ZH: Zhuhai; DG: Dongguan; HZ: Huizhou; SZ: Shenzhen.

The PRDUA consists of nine cities in Guangdong Province (Guangzhou (GZ), Shenzhen (SZ), Foshan (FS), Dongguan (DG), Zhongshan (ZS), Zhuhai (ZH), Huizhou (HZ), Jiangmen (JM), and Zhaoqing (ZQ)), with a total area of 54,770 km^2^ (Figure 1). At the end of 2019, the PRDUA had a permanent population of 64.4689 million, a gross domestic product (GDP) of 868.905 billion RMB, an urban population of 55.622 million, and an urbanization rate of 86.28% [29]. The PRDUA has a high level of economic development and a growing demand for green recreational space. The area is surrounded by mountains on three sides, and contains three rivers that lead to the sea. The topography is undulating, with hills in the west, the Pearl River Delta plain in the middle, and the bay area in the east. The main and tributary streams (Xijiang, Dongjiang, and Beijiang) converge at the mouth of the Pearl River, with dense river networks, crisscrossing rivers, large and small rivers dotted with branches, and diverse types of wetland resources. The GZ-FS-ZS-ZH and GZ-DG-SZ areas surrounding the Pearl River Estuary are highly concentrated, and the built-up area has continuously expanded, which has imposed a serious reduction effect on the ecosystem, resulting in the high floor-area ratio of the river bank, mixed functions, and a lack of waterfront recreational space. A review of the historical evolution process and mechanism of the coupled ELR and recreational use under these circumstances is important for designing an effective approach to combine ELR and RUoEL in the future, which will greatly improve the construction of the recreational bay area.

### 2.2. Data Sources

#### 2.2.1. Land Use and Influence Factor Data

The land use data are the 30-m resolution land cover/use dataset for the Guangdong-Hong Kong-Macao Greater Bay Area from Feng et al. [30], which have a classification accuracy of 0.92 ± 0.02 for ecological land. We obtained an accuracy of 0.94 ± 0.05 by extracting the land use data of the study area from 1990–2017, which were verified by field research. The ELE, Slope, Pre and Tem values were obtained from the former Resource and Environment Science and Data Center (https://www.resdc.cn, accessed on 19 July 2021); the POP data were obtained from Worldpop (https://www.worldpop.org/, accessed on 19 July 2021), and the KDX data were based on the main roads (railroads, national roads, provincial roads, highways) extracted from the traffic atlas. The spatial resolution of all of the above data is 1 km. The GDPPC data were obtained from the Guangdong Provincial Statistical Yearbook, and the statistics were based on county-level administrative regions (except Dongguan). All of the data were spatially analyzed in a 3 × 3 km^2^ grid.

#### 2.2.2. Spatial Data of the RUoEL

Ecological land refers to land space characterized by natural attributes, with the main function to provide ecological services or ecological products, including forests, grasslands, wetlands, rivers, lakes, tidal flats, shorelines, oceans, wastelands, deserts, glaciers, and islands without residents [31]. The RUoEL involves certain transformations and utilizations to make ecological land accessible, and recreational functions to allow it to become an ecological recreational space under the premise of protecting and developing basic ecosystem services (e.g., regulation, supply, support) in the area. At the urban scale, ecological recreational spaces include urban parks, botanical gardens, forest parks, wetland parks, community green spaces, and waterfront open spaces, amongst others [32]. In view of the large area of the PRDUA, the representativeness and criticality of the ecological recreational space patches in the regional ecosystem cultural services, and the data availability, the ecological recreational space of the urban agglomeration does not consider micro-scale features such as streets/community green spaces. Instead, eight types with regional recreational service significance were selected: forest parks, scenic spots, geological parks, water conservancy scenic spots, wetland parks, national A-level ecological scenic spots, urban parks, and country parks. The first six categories are all natural recreational space systems established under the leadership of the relevant ministries and commissions of the State Council of China, and are the main body of China’s ecological recreational space. The seventh category of parks and green spaces mainly refers to large-scale and comprehensive urban parks and specialized parks with high recreational value. The eighth category of country parks has become increasingly popular among residents as an important carrier of leisure tourism in urban agglomerations. Country parks are therefore listed separately from urban parks and forest parks. Community- and township-level forest parks, wetland parks, and 1A-level scenic spots are not included in the scope of this research due to their relatively low cultural service value at the scale of the entire urban agglomeration.

**Figure 2:**
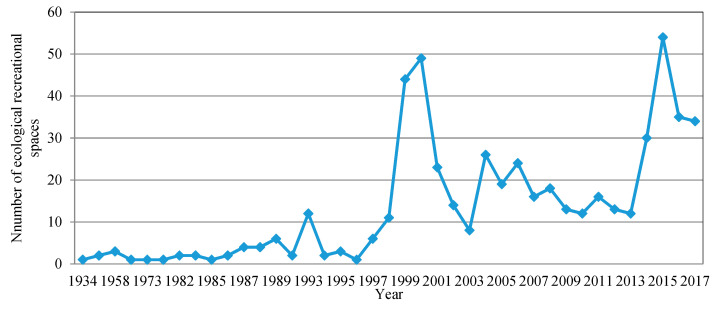
Number of ecological recreational spaces as a function of time.

The spatial information (e.g., name, grade, area) of the ecological recreational space was obtained from public websites of the former Guangdong Provincial Department of Land and Resources, the former Guangdong Provincial Tourism Bureau, the former Guangdong Provincial Forestry Department, and the Ministry of Water Resources, as well as various ecological recreational space directories. Data on a total of 539 ecological recreational spaces were collected by merging multi-name ecological recreational spaces into one place and selecting the larger records in terms of their area and grade. The attribute information in terms of the establishment time and area of various ecological recreational spaces was obtained from the Pearl River Delta Region Forest Park Construction and Development Plan (2010–2020), Baidu Baike; the official government website and government information disclosure application, Sina, Sohu; and other website news [33] (Figure 2).

GPS positioning and visual interpretation were combined to digitally process the ecological recreational space data. Geographic coordinate information was collected at each ecological recreation point, and was imported into ArcGIS to obtain a vector point layer. Combined with the Google image map and point location, the ecological patch where the point is located was digitized to outline the ecological recreational space patch. According to this method, 539 ecological recreational spaces up to 2017 were digitized to obtain a vector diagram of the RUoEL patch distribution. The year 1990 was identified as the inflection point of urban land expansion between the initial stage and rapid growth, and the accelerated growth stage in China [34], and large-scale ecological land restoration in the PRDUA also started around 1986 [35]. Therefore, we choose 1990 as the starting point for the time series analysis of ELR and RUoEL.

### 2.3. Methods

#### 2.3.1. ELR Identification

The ELR patterns in the PRDUA were classified into infilling, edge-expansion, and outlying types based on the spatial expansion characteristics of the local ecological land patches [36,37]. Based on the above definitions, the landscape expansion index (LEI) proposed by Liu et al. (2010) was used to characterize the ELR spatial pattern in detail by defining the spatial relationship between the target patches and the existing patches [37]:

(1) $$LEI=\frac{A_{0}}{A_{0}+A_{v}}\times 100$$

where *A*_0_ is the intersection between the buffer zone and occupied category, and *A_v_* is the intersection between the buffer zone and the vacant category. According to this definition, the value of the *LEI* varies in the range from 0 to 100. *ELR* is defined as infilling when *LEI* > 50, edge-expansion when 0 < *LEI* ≤ 50, and outlying when *LEI* = 0.

#### 2.3.2. Geographically and Temporally Weighted Regression

ELR in urban agglomerations is driven by a combination of natural and socioeconomic factors. This study selected quantitative factors including RUoEL, elevation (ELE), slope (Slope), average annual rainfall (Pre), average annual temperature (Tem), population density (POP), GDP per capita (GDPPC), the shortest distance to a road (KDX), and land urbanization rate (LUR) as the independent variables based on previous findings and data availability. The study area was divided into a 3 × 3 km^2^ grid, and a geographically and temporally weighted regression (GTWR) was used to test the spatial effects of the RUoEL and other factors on ELR.

In contrast to the widely used geographically weighted regression (GWR), which only considers the effect of space when estimating independent variable coefficients, the GTWR can capture the spatiotemporal heterogeneity of variable data [38]. A GTWR model was therefore used in this study to examine the spatiotemporal differentiation of the factors that influence the ecosystem services. The basic GTWR formula is given as:

(2) $$y_{i}=\beta_{0}\left(u_{i},v_{i},t_{i}\right)+\overset{q}{\underset{k=1}{\sum }}\beta_{k}\left(u_{i},v_{i},t_{i}\right)X_{ik}+\varepsilon_{i}$$

where *y_i_* is the area of ELR; *β*_0_(*u_i_*, *v_i_*, *t_i_*) is the regression intercept; *β_k_*(*u_i_*, *v_i_*, *t_i_*) is the regression coefficient; (*u_i_*, *v_i_*, *t_i_*) is the space–time coordinate of research unit *k*; *u_i_*, *v_i_*, and *t_i_* are the latitude, longitude, and data time, respectively; *q* denotes the number of variables; *X_ik_* is the data of variable *q* from research unit *k*; and *ε_i_* is the error term for research unit *k*. The *β_k_*(*u_i_*, *v_i_*, *t_i_*) values for each variable *k* and spatiotemporal location *i* were estimated as follows:

(3) $$\beta \left(u_{i},v_{i},t_{i}\right)={\left[X^{T}W\left(u_{i},v_{i},t_{i}\right)X\right]}^{-1}X^{T}W\left(u_{i},v_{i},t_{i}\right)Y$$

where *W*(*u_i_*, *v_i_*, *t_i_*) = diag (*i*_1_, *i*_2_,…,*i_n_*) is the spatiotemporal weight matrix calculated by the spatiotemporal distance and its decay functions. In this study, we selected the Gaussian function as the spatiotemporal weight matrix function and used cross-validation to determine the adaptive bandwidth, and the established criteria are the corrected Akaike information criterion (AICc), Mean Absolute Error (MAE), the Mean Squared Error (MSE) and goodness-of-fit (R^2^), to assess the model performance.

## 3. Results

### 3.1. Spatial Coupling of Regional Greenways and ELR

A greenway is a sustainably used network of land that contains linear elements, which are planned, designed, and managed for ecological, recreational, cultural, and aesthetic purposes using five key concepts: linear configuration, connectivity, multifunctionality, sustainability, and integration [39]. By January 2011, the PRDUA had built 2372 km of regional (provincial) greenways, linking more than 200 scenic spots, nature reserves, forest parks, country parks, wetland parks, and historical and cultural sites and relics [40], which are equipped with recreation service facilities including multiple recreation functions (e.g., ecological sightseeing, sports, environmental education, cultural experience). The regional greenway is the backbone of the greenway network in the PRDUA, and serves as an important ecological corridor in the region. City governments should promote the construction of various parks and green belts along the greenway network to form an urban ecological network, which may promote the protection and restoration of the ecological environment along the route [41].

**Figure 3:**
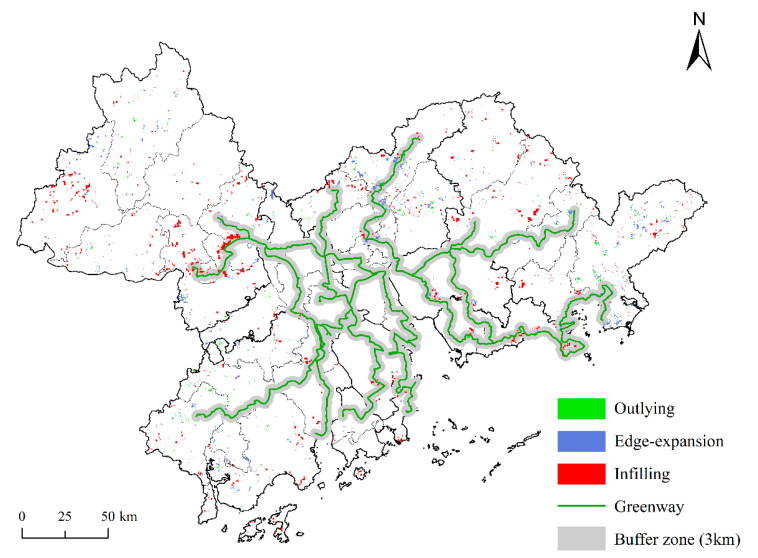
Ecological land restoration patterns within the 3-km buffer zone of the greenway.

This study uses the buffer zone analysis method to verify the effect of the regional greenway construction on the increase of the ELR area on both sides (Figure 3). In 2017, the area of ELR patches within the 1-km buffer zone of the PRDUA regional greenway was 88.39 km^2^, accounting for 8.37% of the total ELR area (1055.55 km^2^). The areas of outlying, edge-expansion, and infilling restoration were 3.95 km^2^ (4.47%), 16.24 km^2^ (18.37%), and 38.30 km^2^ (77.16%), respectively. The area of ELR patches within the 2-km buffer zone of the greenway was 156.28 km^2^, accounting for 14.81% of the total area, with 6.27 km^2^ (4.01%), 34.08 km^2^ (21.81%), and 115.93 km^2^ (74.18%) being restored by outlying, edge-expansion, and infilling restoration areas, respectively. The area of ELR patches within the 3-km buffer zone of the greenway was 210.73 km^2^, accounting for 19.96%, of which 9.65 km^2^ (4.58%), 63.01 km^2^ (29.90%), and 138.07 km^2^ (65.52%) were restored by outlying, edge-expansion and infilling restoration areas, respectively. These results indicate that the ELR area increases with an increasing buffer distance, and that it has a tendency to cluster along the regional greenway. Infilling restoration accounts for a larger proportion mainly because the greenways were generally built along forests, rivers, and roads, and the ecological space area is wider on both sides.

### 3.2. Spatial Coupling of the RUoEL and ELR

**Figure 4:**
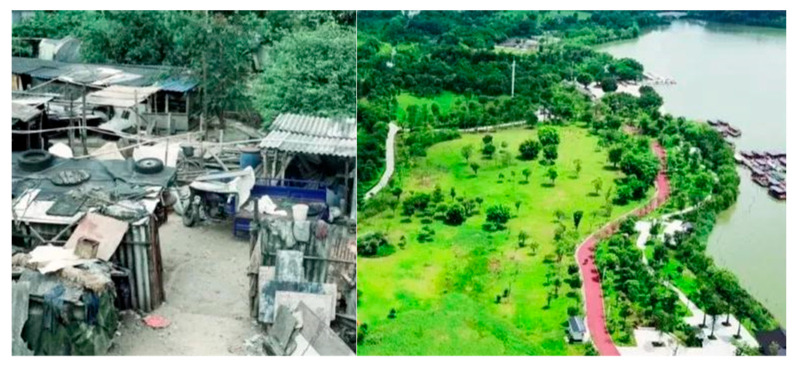
Landscape before and after ELR in Huayang Lake National Wetland Park. Note: In the process of ELR, the government guided the withdrawal of approximately 1,333,333 m^2^ of industrial land in the surrounding area; demolished 223 livestock farms totaling 165,000 m^2^, and promoted the expansion of ecological land and the improvement of recreational facilities. Now, it has become an eco-tourism area. Source: Guangdong Provincial Department of Natural Resources (https://mp.weixin.qq.com/s/4WvcqHufzXvFMeQlUWZRlA, accessed on 17 September 2021).

The Great Greening Campaign of Guangdong Province dates back to 1986 [35]. In August 2013, Guangdong Province declared the “Decision on Comprehensively Promoting a New Round of Greening Guangdong Campaign”. Statistical information indicates that, by the end of 2017, Guangdong Province had built 9809 km of ecological landscape forest belts, completed a carbon sink afforestation of 10,020 km^2^, constructed forest-encircling cities, added 588 forest parks and 132 wetland parks, and performed rural greening in 11,778 villages [42]. The greening of Guangdong promoted the construction of ELR and ecological recreation spaces; such cases include Huayang Lake National Wetland Park in Dongguan City (Figure 4).

**Figure 5:**
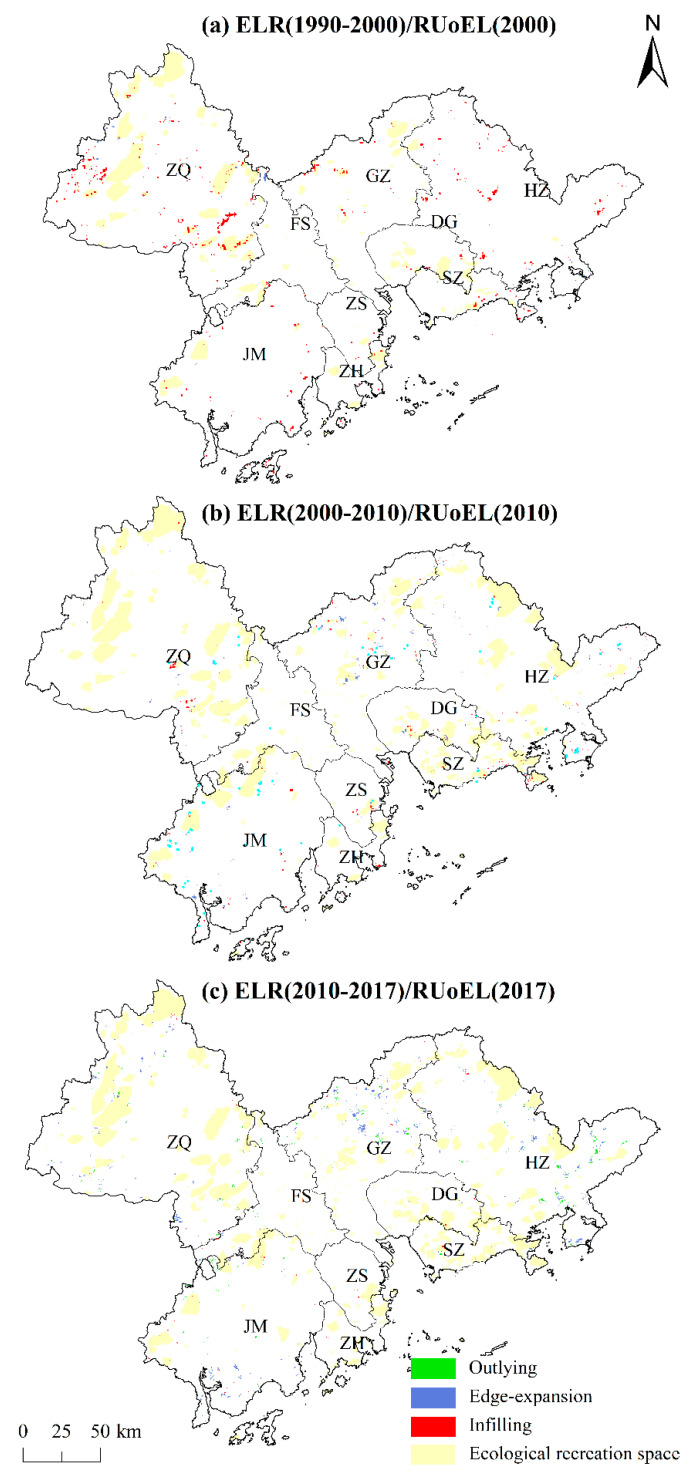
Spatial relationship between the ecological land restoration (ELR) patches and the recreational use of ecological land (RUoEL).

An overlay of the ecological open-space patch elements and ELR patch elements (Figure 5) shows that the two have a certain degree of spatial overlap in each city, especially in the urban contiguous area around the Pearl River Estuary, consisting of four cities (SZ, DG, ZH, and ZS), where the overlap is highly visible, which indicates that ELR under a recreation orientation is more prominent in this area. In 2000, the ecological recreation land was mainly concentrated in the western and northern areas of the PRDUA (e.g., ZQ, northern GZ, and western JM). From 1990 to 2000, ELR was mainly based on infilling restoration, and was mostly concentrated in the forest areas of the forest park agglomeration in southeastern ZQ, forest areas in the west, and near the forest parks of Rangke Mountain and Dachong Reservoir in southeastern SZ. Although there were more ELR patches in HZ, the RUoEL was slower. This indicates that the trend of the internal restoration of large ecological recreation spaces and their surrounding environments was more prominent in the study area.

In 2010, the ecological recreation land in the study area significantly expanded in the SZ-DG region, northern GZ, and south–central ZQ. From 2000 to 2010, the ELR included mainly infilling restoration and edge-expansion. Infilling restoration was sporadically distributed in the ZQ Jinzhongshan Forest Park, DG Dalingshan Forest Park, and SZ Dapeng Peninsula National Geopark, while edge restoration was mostly distributed along the Maodun Lake Forest Park and Yuntai Mountain in northern GZ.

In 2017, the expansion of ecological recreation spaces was small, and only occurred in central GZ, central HZ, and the southern regions. From 2010 to 2017, the ELR was dominated by outlying restoration and edge-expansion restoration, mainly distributed in the edge areas of the study area. The outlying restoration was concentrated in the central HZ Xiangtoushan Forest Park and Longshan Forest Park, while the edge restoration was concentrated along the Liuxi River Forest Park in Jiulongtan Forest Park in central GZ, as well as in the central HZ Yujingfeng Forest Park and the Guzhai Forest Park.

**Figure 6:**
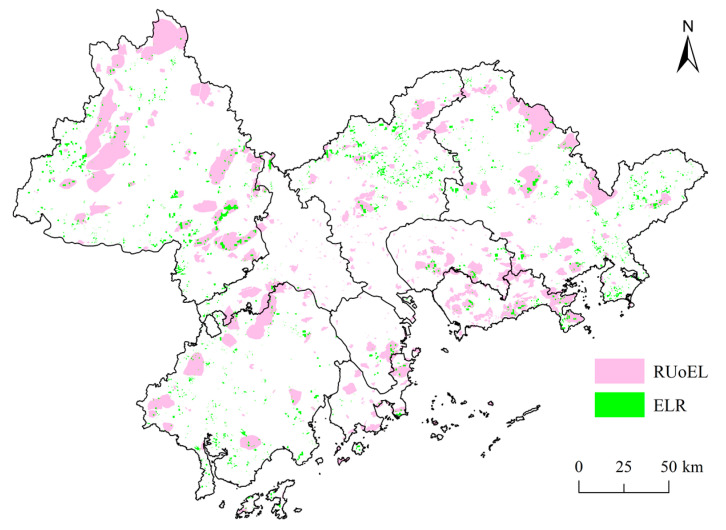
Spatial distribution of the ecological land restoration (ELR) patches and the recreational use of ecological land (RUoEL).

An integration of the spatial evolution patterns of the RUoEL and ELR from 1990 to 2017 (Figure 6) shows that the two overlap to different degrees in each city, especially in the belt around the Pearl River estuary composed of SZ, DG, ZH and ZS. The overlap dynamics of the two are very notable, which indicates that ELR under recreation-oriented use is more prominent in this belt. In the built-up area, the city government constructed green areas with recreational and ecological functions, using urban ELR to improve the living environment, and thus the overlap between ELR and recreational use is high. The RUoEL and ELR patches also largely overlap in eastern HZ, southeastern ZQ, northern FS, and northwestern GZ. In terms of the individual RUoEL, the SZ Dapeng Peninsula National Geological Park, DG Dalingshan Provincial Forest Park, HZ Bailin Lake Water Conservancy Scenic Area, FS Sanshui District Dakeng County Forest Park, GZ Tianlu Lake Provincial Forest Park, and other ecological recreation space patches all have larger areas of infilling and edge-expansion ELR, which indicates that the government adopted afforestation, wetland restoration, and other means to optimize the ecological recreation systems. In particular, the large action of the greening of Guangdong positively promoted ELR, and many ELR and RUoEL construction projects have been simultaneously carried out.

### 3.3. Spatial Effects of the RUoEL on ELR

The R^2^ for the GTWR results is 0.57, and the AIC, MSE, and MAE are 602.814, 0.015, and 102.338, respectively, indicating that the model has a good fitting effect. According to the regression coefficients (Table 1), the RUoEL was found to have a significant positive effect on ELR, indicating that the recreation factor is one of the drivers of ELR in the PRDUA. In addition, ELE, POP, and LUR had a negative effect on ELR because higher elevation areas have higher vegetation cover and less area for ecological restoration, whereas densely populated areas and areas with higher urbanization levels have higher land prices, which makes it more difficult to implement ELR. The Slope, Pre, Tem, GDPPC, and KDX results showed a positive effect on ELR. This indicates that areas with a higher slope and good rain and heat conditions can help promote ELR, whereas a higher GDPPC level indicates a higher demand for recreation quality from residents and higher expectations for ecological quality, thus driving ELR. KDX also helps drive ELR because areas closer to roads have a higher potential for ecological damage and therefore a larger area for ecological restoration.

It is worth noting that the mean, median, and quartiles of the RUoEL were positive, indicating that more than 50% of the regression coefficients were positive. The effect of the RUoEL on ELR was positive in general, but there were a few regression coefficients with negative low values in some local areas. All of the statistical results for TEM, KDX, and GDPPC were positive, indicating that all of the regression coefficients for these three variables were positive at the regional scale. The mean values of the regression coefficients of Slope and Pre were also positive, but there were a few negative Slope regression coefficients, indicating that the effect was negative in some local areas. The median Pre value was less than 0, indicating that the regression effect was negative in more than 50% of the areas. The mean values of the ELE, POP, and LUR regression coefficients were less than 0, indicating that the overall effect was negative in most areas.

**Figure 7:**
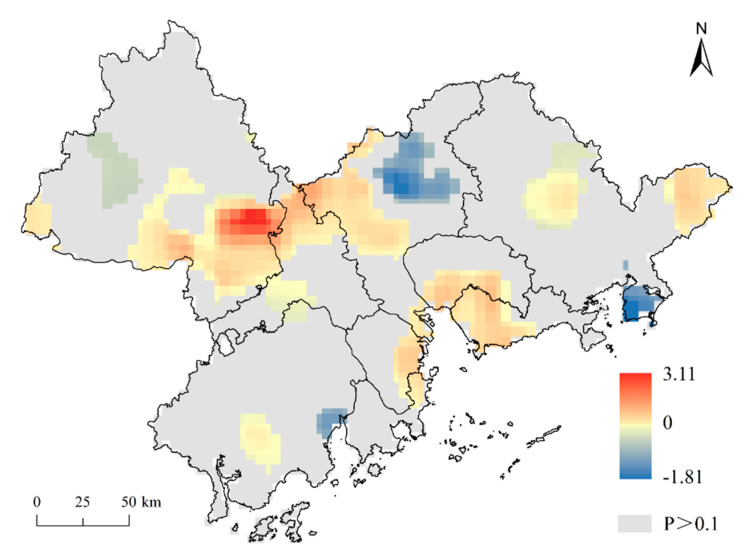
Spatial distribution of the regression coefficients (*p* ≤ 0.1) of the recreational use of ecological land (RUoEL) on the ecological land restoration (ELR).

Further spatial analysis of the regression coefficients (Figure 7) showed that the RUoEL had a significant positive impact on ELR in SZ, DG, ZH, and ZS, as well as in the eastern part of HZ, southeastern ZQ, northern FS, and northwestern GZ. These areas have large ecological recreation spaces or are located close to densely populated built-up areas, which facilitates the formation of recreation-oriented ELR. In GZ, some of the RUoEL in administrative boundary areas (e.g., Conghua District, Zengcheng District, the southern peninsula of Huidong County, Fengkai County, and Huaiji County) had a negative impact on ELR. This is because these areas are located far from built-up areas and high-grade ecological open spaces; thus, the restoration of basic rivers, vegetation, and wetlands is the main focus, and there is insufficient incentive to restore ecological land oriented for the construction of ecological recreation spaces.

## 4. Discussion and Implications

### 4.1. Discussion

There is a certain degree of spatial coupling between the ELR and RUoEL distribution, which is similar to the finding of Stanford et al. [9] that although more than half (54%) of the ecological restoration projects are for ecological purposes, a certain proportion (around 20%) of the projects are carried out for human interests (e.g., aesthetics, recreation, education). Specifically, a spatial correlation is observed between the regional greenway construction and ELR. Regional greenways have a high urban ELR frequency, and are concentrated in urban, built-up areas, which causes the regional greenways and ELR to generate spatial clusters. It is indicated that regional greenways promote the restoration of ecological land along the route. The positive effects of greenways in the PRDUA in terms of ecosystem improvement have also been verified in practical cases in Central Europe. Approximately 15% of the landscape in Central Europe is covered by a green infrastructure network, which provides a wide range of ecosystem services. Among them, cultural services (e.g., with entertainment and aesthetic values) are also relatively abundant because they are harmonious with natural and near-natural ecosystems under good environmental conditions [43]. The spatial mapping of RUoEL and ELR shows that the two are spatially dependent, especially in the towns and densely populated areas near the Pearl River Estuary, indicating that the spatial coupling may be related to socio-economic factors such as population and the urbanization level.

The change of the RUoEL area showed a significant positive impact on the change of the ELR area, indicating that recreational use is a driving factor for ELR in the PRDUA. There are three main reasons for this driving effect. First, the government has increased green open space in urban built-up areas, which means that the construction of urban natural recreational space drives the restoration of urban ecological land [44]. The second reason is the need to improve the recreational quality of existing ecological land through restoration [45]. The third reason is the implementation of the recreation-oriented restoration of abandoned land to establish wetland parks, forest parks, and other recreational areas [46]. Altitude, population density, and the land urbanization rate have a negative impact on ELR. Higher-altitude areas have higher vegetation coverage and less ELR area, and densely populated areas and areas with higher levels of urbanization have higher land prices, resulting in a greater resistance to ELR implementation, thus reducing the ELR area. Slope, Pre, Tem, GDPPC, and KDX have a positive effect on ELR. Areas with large slopes and good rain and heat conditions help promote ELR. Higher GDPPC is generally associated with higher residential expectations for the quality of the ecological environment and thus is a greater driving force for the ELR. Areas located near highways also have a greater risk of suffering from ecological damage, and thus contribute to larger ecological land areas that can be restored. The results suggested that ecological land transitions in terms of the spatial–temporal evolution of the dominant morphology (i.e., ELR) and recessive morphology (i.e., RUoEL), and their interactive characteristics, are driven by a combination of natural and human environments.

The results also indicate spatial differences in the impact of the RUoEL on the ELR in the PRDUA. The coupled relationship between the RUoEL and ELR is more notable in areas with high-level ecological recreational spaces or in densely populated built-up areas because these areas are likely to form recreation-oriented ELR. RUoEL has had a significant positive impact on the ELR in the area around the Pearl River Estuary, including SZ, DG, ZH, and ZS, as well as the east of Huidong County in HZ, the junction of Huicheng District and Boluo County, southeastern ZQ, northern FS, and northwestern GZ, indicating that the recreation-oriented restoration of ecological land is the main type of ecological restoration in these areas. These areas have large-scale ecological recreational spaces, or are located near densely populated built-up areas, which facilitates recreation-oriented ecological restoration. In contrast, the RUoEL has a negative impact on the restoration of ecological land in other areas (e.g., Guangzhou’s Conghua District, Zengcheng District, the southern peninsula of Huidong County, and some areas at the junction of Fengkai and Huaiji counties), indicating that recreation-oriented ELR is not the main type of ELR in these areas. The main reason is that these areas are located far from built-up areas and high-level ecological recreational spaces, which leads to the predominance of the restoration of basic rivers, vegetation and wetlands, and ELR driven by the construction of ecological recreational spaces is comparatively insufficient.

### 4.2. Policy Implications

In order to improve ecosystem services and public health, the regional government should promote the coupling of ecosystem preservation and social-cultural functions (e.g., recreational services). It is therefore necessary to understand the coupling relationship between the ecosystem and recreational elements, and to promote the optimization of a recreation-oriented ELR layout according to the suitability of recreational activities. For example, the trend of coupled ELR and RUoEL determined in this study indicates that ELR around the high-level ecological recreation spaces of the PRDUA and surrounding urban built-up areas has great potential, and can be combined with the expansion of recreational functions or the development of the tourism industry to transform restored land into a multifunctional space for ecological conservation and leisure. For this reason, regional governments should pay attention to the overall planning and layout of ELR and its recreational use according to the local conditions.

In addition, according to the coupled rules of RUoEL and ELR, the regional government should coordinate and combine the restoration and utilization of ecological land with the construction of regional ecological leisure/tourism function areas to effectively improve the ecosystem’s structure and environment. The government could specifically improve the quality of recreational space and leisure services in ecological restoration areas, develop internal greenway networks and scenic roads, enhance the agglomeration of ELR and recreational use, and enhance the comprehensive benefits of regional ELR and recreational use. The development of leisure/tourism services will simultaneously promote and narrow the gap of public health service development in marginal areas.

Lastly, the regional government should promote healthy restoration models of ecological corridor systems such as greenways, (i.e., the effective connection and communication medium of isolated ecological patches and scattered ecological land along the corridor), thereby effectively improving the ecological environment and enhancing the environmental quality through ELR. The regional government should further promote the construction of a corridor-based ecological recreation network that organically connects water areas, green spaces, wetlands, forests, and other ecological patches, and configures urban parks and community parks along the corridor to truly integrate ecology into the city, rather than embellishing the city with ecology. The government can also accelerate the restoration of ecological recreational corridors in densely populated areas, expand linear ecological recreational spaces, and simultaneously introduce nature into urban life.

### 4.3. Research Innovation, Limitations and Prospects

With the rapid growth of the population and recreational demand in urban agglomeration areas, there may be a certain relationship between ecological restoration and recreational uses, but this impact has been overlooked compared to other perturbations [10,12]. The innovation of this study is to characterize the spatial distribution of ELR patterns, and to investigate the coupling mechanism of ELR and recreational use. The results reveal their spatial coupling characteristics, as well as the impacts of RUoEL and other socio-economic, environmental factors on ELR, which provides a foundation for regional ELR and recreational use pattern optimization. This article also preliminarily describes the spatial relationship between urban agglomeration greenways and ELR. However, there are some limitations in this study. First, as exploratory research, this study only outlines the spatial coupling characteristics of urban agglomeration recreation and ELR. Follow-up research should therefore further investigate the specific coupling modes of ELR and recreational use through more detailed urban-scale surveys and interviews. This study also only explored the current characteristics and mechanisms. Follow-up research could build an index system of ELR and recreational use suitability, and could comprehensively analyze the ideal pattern of coupling and coordination between RUoEL and ELR. Third, this study analyzes the impact of all types of RUoEL on ELR from the perspective of spatial patterns. In the future, additional attention can be paid to the evolution, planning layout, and operational mode of restoration and recreational use based on local-scale wetlands, mines, forests, grasslands, and coastal zones in order to further reveal the coupling mechanism of site-scale ELR and recreational use.

## 5. Conclusions

The current ELR layout lacks comprehensive planning for the allocation of recreational functions, resulting in low restoration efficiency and poor sustainability in some areas. In addition, previous Chinese ecological restoration studies focused on the restoration of ecological elements (e.g., water, forests, soil) with insufficient consideration of the functional coordination and integrity of the ecosystem. The integrity of an urban agglomeration ecosystem and the relevance of the environmental impact indicate that the regional ecological environment must be considered as a regional community.

This study, therefore, takes the PRDUA as a whole to explore the spatial interaction and mechanism of ELR and RUoEL. To this end, we first identified the evolution of the spatial pattern of ELR and RUoEL (including the recreational use of greenways and ecological land patches) from 1990 to 2017. We then superimposed the restoration and recreational use data to analyze their coupling pattern, and explored the driving mechanism of RUoEL factors in the urban agglomeration on ELR. The results show a certain degree of spatial agglomeration in RUoEL and ecological restoration, indicating that the PRDUA promotes not only ELR but also the recreational use of ecological sites or neighboring areas. The RUoEL is similar to the regional average temperature, road accessibility, GDPPC, Slope, Pre, and other variables, which show generally positive effects on ELR. But the overall effect of altitude, population density and land urbanization on ELR is negative. There are spatial differences in the impact of the RUoEL on the restoration of ecological land in the PRDUA. The RUoEL has had a significant positive impact on ELR in densely populated areas around the Pearl River Estuary composed of Shenzhen, Dongguan, Zhuhai, and Zhongshan, the suburbs of the Guangzhou-Foshan-Zhaoqing metropolitan area, and the high-level ecological recreational space gathering areas in the outer suburbs of Huizhou. This indicates a notable integration trend of ELR and RUoEL in these areas.

However, this study only outlines the spatial coupling characteristics and mechanisms of recreation and ELR in urban agglomerations. Subsequent research should further investigate the coupling of ELR and RUoEL at the local and site scales of urban agglomerations, and should comprehensively analyze the ideal pattern of coupling between RUoEL and ELR to provide policy recommendations for their coordinated layout in urban agglomerations.

## Figures and Tables

**Table 1:** Descriptive statistics of each variable.

Variables	Mean	STD	Min	Median	Max
Intercept	−0.103	0.007	−0.113	−0.105	−0.090
RUoEL	0.223	0.469	−1.810	0.133	3.114
ELE	−0.007	0.005	−0.015	−0.007	−0.000
Slope	0.170	0.273	−1.861	0.158	1.225
Pre	0.022	0.696	−6.574	−0.006	2.602
Tem	0.102	0.010	0.089	0.098	0.121
POP	−0.050	0.002	−0.055	−0.049	−0.048
GDPPC	0.027	0.001	0.024	0.027	0.029
KDX	0.026	0.006	0.014	0.023	0.042
LUR	−0.042	0.004	−0.049	−0.042	−0.035

## Data Availability

Not applicable.
